# FDA approvals of specialty drugs, 2000-2024

**DOI:** 10.1093/haschl/qxag035

**Published:** 2026-02-07

**Authors:** Sophie E Knox, Claire H Brennan, Daniel E Enright, Peter J Neumann, James D Chambers

**Affiliations:** The Center for the Evaluation of Value and Risk in Health, Institute for Clinical Research and Health Policy Studies, Tufts Medical Center, Boston, MA 02111, United States; The Center for the Evaluation of Value and Risk in Health, Institute for Clinical Research and Health Policy Studies, Tufts Medical Center, Boston, MA 02111, United States; The Center for the Evaluation of Value and Risk in Health, Institute for Clinical Research and Health Policy Studies, Tufts Medical Center, Boston, MA 02111, United States; The Center for the Evaluation of Value and Risk in Health, Institute for Clinical Research and Health Policy Studies, Tufts Medical Center, Boston, MA 02111, United States; The Center for the Evaluation of Value and Risk in Health, Institute for Clinical Research and Health Policy Studies, Tufts Medical Center, Boston, MA 02111, United States

## Introduction

Specialty drugs, used to treat complex conditions such as cancer, autoimmune disorders, and rare diseases, including both biologics and high-cost small molecule therapies, represent less than 5% of prescriptions yet account for 54% of pharmaceutical spending (approximately $263 billion in 2024), up from 47% in 2019. The median annual list prices of medicines launched in 2024 exceeded $350,000, with oncology and rare disease therapies surpassing $400 000 per patient.^1^

Rising specialty drug spending raises concerns about the sustainability of health care budgets, yet prior studies have largely focused on costs and utilization trends, offering limited insight into the extent to which growth in specialty spending reflects a structural shift in the composition of newly approved therapies.^2,3^

To address this gap, we examined US Food and Drug Administration (FDA) approvals of specialty drugs from 2000 to 2024 to estimate the share of therapies classified as specialty medicines and to characterize their key attributes, including orphan status and therapeutic area.

## Methods

We identified novel drug and biologic approvals from 2000 to 2024 in the FDA's New Molecular Entity database^4^ and excluded drugs withdrawn from the market (Appendix Table S1). Because the FDA does not categorize approvals by specialty status, we assigned specialty designations using the specialty drug lists (as of September 2025) from the 3 largest pharmacy benefit managers (PBMs): CVS Caremark, Express Scripts, and OptumRx. We considered a drug a specialty drug if it appeared on at least one PBM list. PBM specialty lists are directly relevant to payer and patient experience, as they reflect payer recognition of a product's complexity, cost, and management intensity through benefit design, cost sharing, and utilization management. We classified FDA-approved cell and gene therapies as specialty due to their costs and management requirements.

For each specialty therapy, we categorized all initial and follow-on indications by orphan status, cancer indication, and FDA expedited review pathway. We used Poisson regression to test temporal trends in annual specialty drug approvals, adjusting for total annual approvals via a log offset.

## Results

**Figure 1.**
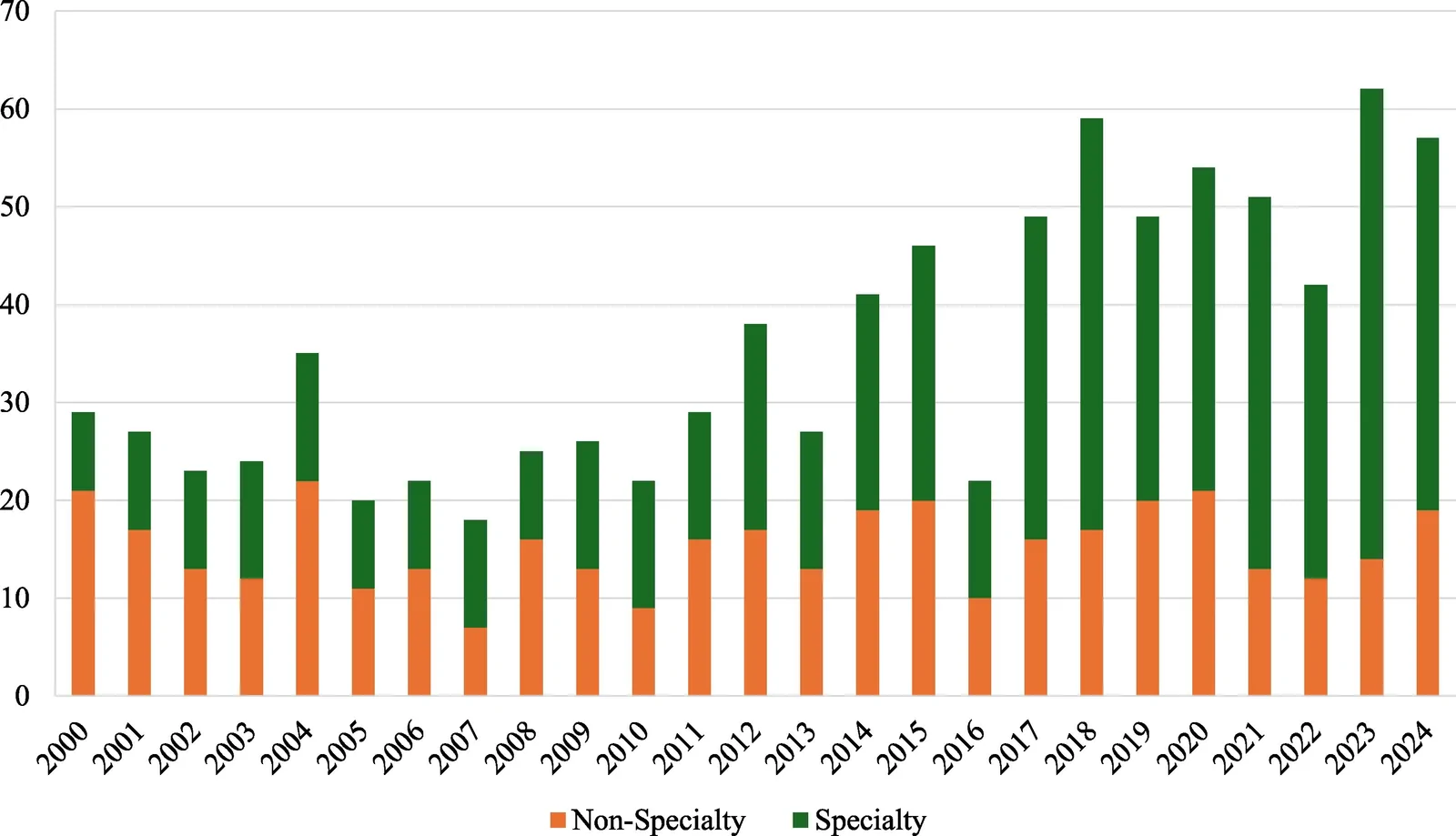
FDA drug approvals by year, categorized by specialty status.

Between 2000 and 2024, the FDA approved 897 novel drugs, of which 516 (57%) were classified as specialty. Specialty approvals increased at approximately 3% per year (β = 0.0295, *P* < .001) (Figure 1). Since 2012, specialty drugs have accounted for more than half of FDA drug approvals in every year.

Including follow-on approvals, the FDA approved 947 specialty drug-indication pairs (Appendix Table S2). Among these therapies, 188 (36%) received at least one follow-on indication, with a mean of 1.7 (standard deviation 1.8) per therapy. Among specialty drug–indication pairs, 55% (525) had an orphan designation and 46% (435) were for cancer. The FDA granted expedited reviews to 251 pairs (27%) through one program, 239 (25%) through 2, 122 (13%) through 3, and 14 (1%) through 4 programs.

## Discussion

The FDA has approved significantly more specialty therapies over time, both in absolute number and as a share of all approvals, suggesting a sustained shift in pharmaceutical development toward complex, high-cost treatments.

This pattern likely reflects scientific advances enabling therapies that target genetic, molecular, and biomarker-defined disease mechanisms, particularly in oncology and rare diseases. Federal policies have also shaped specialty drug development through financial incentives, market exclusivity, and expedited review pathways. The Orphan Drug Act (1983) provides incentives and market exclusivity for therapies targeting rare diseases, while the Regenerative Medicine Advanced Therapy designation under the 21st Century Cures Act (2016) grants eligible cell and gene therapies with early FDA guidance, eligibility for accelerated approval based on surrogate endpoints, and priority review to expedite development and regulatory evaluation for serious or life-threatening conditions. More recently, the Inflation Reduction Act (2022) established a longer timeline to Medicare price negotiations for biologics compared with small molecule drugs, reinforcing incentives for their development.

Specialty therapies often represent important advances, and frequently qualify for FDA expedited review, particularly in oncology and rare diseases. However, their high costs and pervasiveness can also create financial strains on patients and the health system, prompting utilization management tools such as patient subgroup restrictions (eg, eligibility based on clinical characteristics or biomarker status) and step therapy protocols (prior treatment failure requirements), which may limit access. Against this backdrop, research has found that US commercial health plan use of UM for specialty therapies is increasing over time.^5^ Our findings underscore the need for policies that balance incentives for innovation with mechanisms to ensure affordability and equitable access as use of specialty therapies continue to expand.

Although our analysis ends in 2024, FDA approvals in 2025 suggest continuity with these longer-term patterns, even as PBM specialty drug lists have not yet fully incorporated all newly approved products. Of the 50 novel drugs approved in 2025, 18 (36%) were biologics, 14 (28%) were oncology therapies, and 26 (52%) received orphan designation; 37 (74%) were approved through expedited regulatory pathways. These approvals largely targeted high-acuity conditions managed within specialty care, reflecting continued innovation in treatments for advanced malignancies and rare immune-mediated, genetic, and neurologic disorders.

This study has several limitations. In the absence of a standardized definition of specialty, we relied on an operational definition of specialty drugs based on current PBM designations, which vary across organizations and may change over time. We did not use the Medicare Part D definition (based on a monthly cost threshold, currently $670) used in other research,^2^ because it would exclude Part B drugs that typically meet the criteria used to categorize specialty therapies. In addition, we did not assess post-approval pricing, coverage, or utilization, which shape the budgetary and access implications of growth in specialty drug approvals.

## Supplementary Material

qxag035_Supplementary_Data
