# Supplementary material for: FDA approvals of specialty drugs, 2000-2024
Source: Health Aff Sch. 2026 Feb 7;4(2):qxag035. doi: 10.1093/haschl/qxag035 (PMC12927497; doi:10.1093/haschl/qxag035)
Supplement: qxag035_Supplementary_Data [file qxag035_supplementary_data.zip › Appendix 2026 01 09.docx]

**Appendix:**

Table 1. Drug Withdrawals 2000-2024

| **Proprietary Name** | **Active Ingredient/Moiety** | **FDA Approval Date** | **Review Designation** | **Date of Withdrawal** | **Reason for withdrawal** |
| --- | --- | --- | --- | --- | --- |
| Amevive | alefacept | 1/30/2003 | Standard | 11/2011 | Poor efficacy |
| Belviq | lorcaserin hydrochloride | 6/27/2012 | Standard | 2/13/2020 | Increased occurrence of cancer in clinical trial |
| Bextra | valdecoxib | 11/16/2001 | Standard | 4/7/2005 | Cardiovascular risk |
| Bexxar | tositumomab, iodine I 131 tositumomab | 6/27/2003 | Priority | 10/23/2013 | Clinical trial failed to demonstrate benefit |
| Erwinaze | asparaginase erwinia chrysanthemi | 11/18/2011 | Priority | 3/31/2023 | License withdrawn by Jazz |
| NeutroSpec | technetium Tc 99m fanolesomab | 7/2/2004 | Standard | 12/2005 | Withdrawn following serious and life-threatening cardiopulmonary events |
| Pepaxto | melphalan flufenamide | 2/26/2021 | Priority | 2/23/2024 | Clinical trial failed to verify benefit |
| Raptiva | efalizumab | 10/27/2003 | Standard | 6/8/2009 | Risk of Progressive Multifocal Leukoencephalopathy |
| Xigris | drotrecogin alfa | 11/21/2001 | Priority | 10/25/2011 | Lack of survival benefit in sepsis treatment |

**References**

1. Amevive; The Dermatologist. *Astellas Pharma US Announces Discontinuation of Manufacturing for Amevive*. Updated January 6, 2012. Accessed September 24, 2025. https://www.hmpgloballearningnetwork.com/site/thederm/content/astellas-pharma-us-announces-discontinuation-manufacturing-amevive
2. Belviq; U.S. Food and Drug Administration. *FDA requests the withdrawal of the weight-loss drug Belviq, Belviq XR (lorcaserin) from the market.* Updated January 14, 2022. Accessed September 24, 2025.

https://www.fda.gov/drugs/fda-drug-safety-podcasts/fda-requests-withdrawal-weight-loss-drug-belviq-belviq-xr-lorcaserin-market

1. Bextra; U.S. Food and Drug Administration. *COX-2 Selective (includes Bextra, Celebrex, and Vioxx) and Non-Selective Non-Steroidal Anti-Inflammatory Drugs (NSAIDs).* Updated February 6, 2018. Accessed September 24, 2025. https://www.fda.gov/drugs/postmarket-drug-safety-information-patients-and-providers/cox-2-selective-includes-bextra-celebrex-and-vioxx-and-non-selective-non-steroidal-anti-inflammatory
2. Bexxar; U.S. Food and Drug Administration. *GlaxoSmithKline LLC; Withdrawal of Approval of the Indication for Treatment of Patients With Relapsed or Refractory, Low Grade, Follicular, or Transformed CD20 Positive Non-Hodgkin's Lymphoma Who Have Not Received Prior Rituximab; BEXXAR.* Updated October 23, 2013. Accessed September 24, 2025. https://www.federalregister.gov/documents/2013/10/23/2013-24840/glaxosmithkline-llc-withdrawal-of-approval-of-the-indication-for-treatment-of-patients-with-relapsed#:~:text=The%20Food%20and%20Drug%20Administration%20(FDA)%20is%20withdrawing%20approval%20of,its%20opportunity%20for%20a%20hearing.
3. Erwinaze; American Society of Health-System Pharmacists. *Drug Shortages - Asparaginase Erwinia chrysanthemi.* Updated June 11, 2024. Accessed September 24, 2025. https://www.ashp.org/drug-shortages/current-shortages/drug-shortage-detail.aspx?id=482
4. NeutroSpec; U.S. Food and Drug Administration*. Information for Healthcare Professionals - [Technetium (99m Tc) fanolesomab] marketed as NeutroSpec (12/2005).* Updated July 17, 2015. Accessed September 24, 2025. https://www.fda.gov/drugs/postmarket-drug-safety-information-patients-and-providers/information-healthcare-professionals-technetium-99m-tc-fanolesomab-marketed-neutrospec-122005
5. Pepaxto; U.S. Food and Drug Administration*. FDA issues final decision to withdraw approval of Pepaxto (melphalan flufenamide)*. Updated February 23, 2024. Accessed September 24, 2025. <https://www.fda.gov/drugs/drug-safety-and-availability/fda-issues-final-decision-withdraw-approval-pepaxto-melphalan-flufenamide>
6. Raptiva; U.S. Food and Drug Administration. *FDA Statement on the Voluntary Withdrawal of Raptiva From the U.S. Market.* Updated December 7, 2015. Accessed September 24, 2025. <https://www.fda.gov/drugs/postmarket-drug-safety-information-patients-and-providers/fda-statement-voluntary-withdrawal-raptiva-us-market>
7. Xigris; U.S. Food and Drug Administration. *FDA Drug Safety Communication: Voluntary market withdrawal of Xigris [drotrecogin alfa (activated)] due to failure to show a survival benefit.* Updated February 9, 2018. Accessed September 24, 2025. <https://www.fda.gov/drugs/drug-safety-and-availability/fda-drug-safety-communication-voluntary-market-withdrawal-xigris-drotrecogin-alfa-activated-due>

Table 2: Novel Specialty Drugs Approved 2000-2024

|  | **All years**  **(2000-2024)** | **2000-2004** | **2005-2009** | **2010-2014** | **2015-2019** | **2020-2024** |
| --- | --- | --- | --- | --- | --- | --- |
| Total drug approvals | 516 | 53 | 51 | 83 | 142 | 187 |
| Total drug-indication approvals | 947 | 112 | 116 | 216 | 262 | 241 |
|  |  |  |  |  |  |  |
| **Drug-indication approvals, No. (%)** | | | | | | |
| Orphan | 525 (55) | 55 (49) | 63 (54) | 116 (54) | 128 (49) | 163 (68) |
| Cancer | 435 (46) | 43 (38) | 46 (40) | 130 (60) | 117 (45) | 99 (41) |
| **Expedited Review program** | | | | | | |
| Priority | 515 (54) | 35 (31) | 55 (47) | 132 (61) | 152 (58) | 141 (58) |
| Fast-track | 198 (21) | 27 (24) | 13 (11) | 26 (12) | 56 (21) | 76 (32) |
| Accelerated | 193 (20) | 21 (19) | 21 (18) | 51 (24) | 41 (16) | 59 (25) |
| Breakthrough^a^ | 245 (26) | 2 (2) | 3 (3) | 58 (27) | 91 (35) | 91 (38) |
| **Number of expedited review programs** | | | | | | |
| 0 | 321 (34) | 62 (55) | 53 (46) | 54 (25) | 87 (33) | 65 (27) |
| 1 | 251 (27) | 23 (21) | 39 (34) | 82 (38) | 58 (22) | 49 (20) |
| 2 | 239 (25) | 19 (17) | 19 (16) | 55 (25) | 72 (28) | 74 (31) |
| 3 | 122 (13) | 8 (7) | 5 (4) | 25 (12) | 42 (16) | 42 (17) |
| 4 | 14 (1) | 0 (0) | 0 (0) | 0 (0) | 3 (1) | 11 (5) |

^a^The FDA breakthrough therapy program was established in 2012. Drugs first approved before 2012 can still qualify when the FDA grants breakthrough designation to a follow-on indication. This happened twice for drugs approved in 2000-2004 and three times for those approved in 2005-2009.
